# Formation of soluble amyloid oligomers and amyloid fibrils by the multifunctional protein vitronectin

**DOI:** 10.1186/1750-1326-3-16

**Published:** 2008-10-21

**Authors:** Thuzar M Shin, J Mario Isas, Chia-Ling Hsieh, Rakez Kayed, Charles G Glabe, Ralf Langen, Jeannie Chen

**Affiliations:** 1 Zilhka Neurogenetic Institute, University of Southern California, Los Angeles, California 90033, USA; 2 Department of Neurology, University of Texas Medical Branch School of Medicine, Galveston, Texas 77555, USA; 3 Department of Molecular Biology and Biochemistry, University of California, Irvine, California 92697, USA

## Abstract

**Background:**

The multifunctional protein vitronectin is present within the deposits associated with Alzheimer disease (AD), age-related macular degeneration (AMD), atherosclerosis, systemic amyloidoses, and glomerulonephritis. The extent to which vitronectin contributes to amyloid formation within these plaques, which contain misfolded, amyloidogenic proteins, and the role of vitronectin in the pathophysiology of the aforementioned diseases is currently unknown. The investigation of vitronectin aggregation is significant since the formation of oligomeric and fibrillar structures are common features of amyloid proteins.

**Results:**

We observed vitronectin immunoreactivity in senile plaques of AD brain, which exhibited overlap with the amyloid fibril-specific OC antibody, suggesting that vitronectin is deposited at sites of amyloid formation. Of particular interest is the growing body of evidence indicating that soluble nonfibrillar oligomers may be responsible for the development and progression of amyloid diseases. In this study we demonstrate that both plasma-purified and recombinant human vitronectin readily form spherical oligomers and typical amyloid fibrils. Vitronectin oligomers are toxic to cultured neuroblastoma and retinal pigment epithelium (RPE) cells, possibly via a membrane-dependent mechanism, as they cause leakage of synthetic vesicles. Oligomer toxicity was attenuated in RPE cells by the anti-oligomer A11 antibody. Vitronectin fibrils contain a C-terminal protease-resistant fragment, which may approximate the core region of residues essential to amyloid formation.

**Conclusion:**

These data reveal the propensity of vitronectin to behave as an amyloid protein and put forth the possibilities that accumulation of misfolded vitronectin may contribute to aggregate formation seen in age-related amyloid diseases.

## Background

Vitronectin is a multi-functional glycoprotein involved in a variety of physiological processes. It is present in blood at a concentration of 0.2–0.45 mg/ml, constituting 0.1–0.5% of plasma protein, and is a component of the extracellular matrix [1,2]. While the liver is the primary site of vitronectin synthesis, several extrahepatic sites have been reported, including the retina [3-5], brain [6], and vascular smooth muscle cells [7]. The multi-functional properties of vitronectin are mediated by its ability to interact with many other macromolecules. Vitronectin inhibits fibrinolysis through its N-terminal somatomedin B (SMB) domain, which binds to and stabilizes type 1 plasminogen activator inhibitor (PAI-1) [8,9]. Cell adhesion, spreading, and migration is promoted by the interaction of vitronectin's RGD sequence with several integrin receptors, including the αvβ3 vitronectin receptor [10]. Vitronectin associates with components of the extracellular matrix via a collagen-binding domain and a polycationic heparin-binding domain [11,12]. The C-terminal heparin-binding domain also prevents complement-mediated cell lysis by inhibiting assembly of the C5b-C9 membrane attack complex and blocking perforin pore formation [13-15].

Whilst its role in maintaining homeostasis has been studied extensively, the role of vitronectin in disease is not well understood, though it has been implicated in a number of disease processes. For example, vitronectin expression is upregulated in animal models of acute and chronic inflammation [16] and in fibrotic tissues [17,18]. In addition, serum levels of vitronectin are elevated in patients with atherosclerosis [19], type 2 diabetes [20], and Alzheimer disease (AD) [20]. Vitronectin has been identified in deposits associated with AD, atherosclerosis, systemic amyloidoses, and glomerulonephritis [21-33]. In addition, we and others [4,5,26,27,34-36] have observed vitronectin reactivity in all drusen samples analyzed, which are extracellular ocular deposits associated with aged eyes and eyes with age-related macular degeneration. Accumulation of vitronectin in extracellular deposits may be related to its proclivity to undergo structural rearrangements and its tendencies to self-associate and form multimers and high molecular aggregates in vitro, even under near physiological conditions [37-39]. The structural basis for the tendency of vitronectin to aggregate is incompletely understood. It has been observed that these insoluble vitronectin-containing disease deposits exhibit thioflavin staining [40-42], indicating an underlying process of protein misfolding and amyloid formation. In this study we address whether formation of amyloid structures may be a product of vitronectin misfolding. The investigation of vitronectin aggregation is significant since the formation of spherical and protofibrillar oligomers, as well as fibrils, are common properties of amyloid proteins, although they share neither sequence nor native structural homology. A growing body of evidence indicates that soluble prefibrillar oligomers may be the primary pathogenic species in amyloidopathies [43-47]. Thus, if vitronectin does indeed form amyloid oligomers or fibrils, its misfolding may contribute to the pathophysiology of the aforementioned diseases.

The development of the conformation-specific A11 antibody which recognizes soluble nonfibrillar oligomers made from a number of amyloidogenic peptides and proteins, but not monomers or fibrils, has aided the analysis of these toxic aggregates [48]. This antibody facilitates the characterization of soluble oligomers as markers of amyloid diseases and enables the identification of novel amyloid diseases wherein there is oligomer accumulation without abundant fibril deposition. One such disease is desmin-related cardiomyopathy [49,50]. We recently reported that human ocular drusen contain nonfibrillar oligomers, which suggests that age-related macular degeneration may be another example of this type of amyloidosis [35], and a number of studies identify vitronectin as one of the most abundant drusen proteins [4,26,34,51]. Vitronectin is an appealing candidate for misfolding due to its widespread distribution in the body and its association with insoluble, disease-associated plaques. In this study, we demonstrate that vitronectin behaves as an amyloid protein *in vitro *and soluble nonfibrillar vitronectin oligomers are toxic to cultured cells. Amyloidogenic propensity and toxicity suggest that vitronectin misfolding and aggregation may contribute to the pathophysiology of age-related diseases.

## Results

### Vitronectin deposits in senile plaques of Alzheimer brain

**Figure 1:**
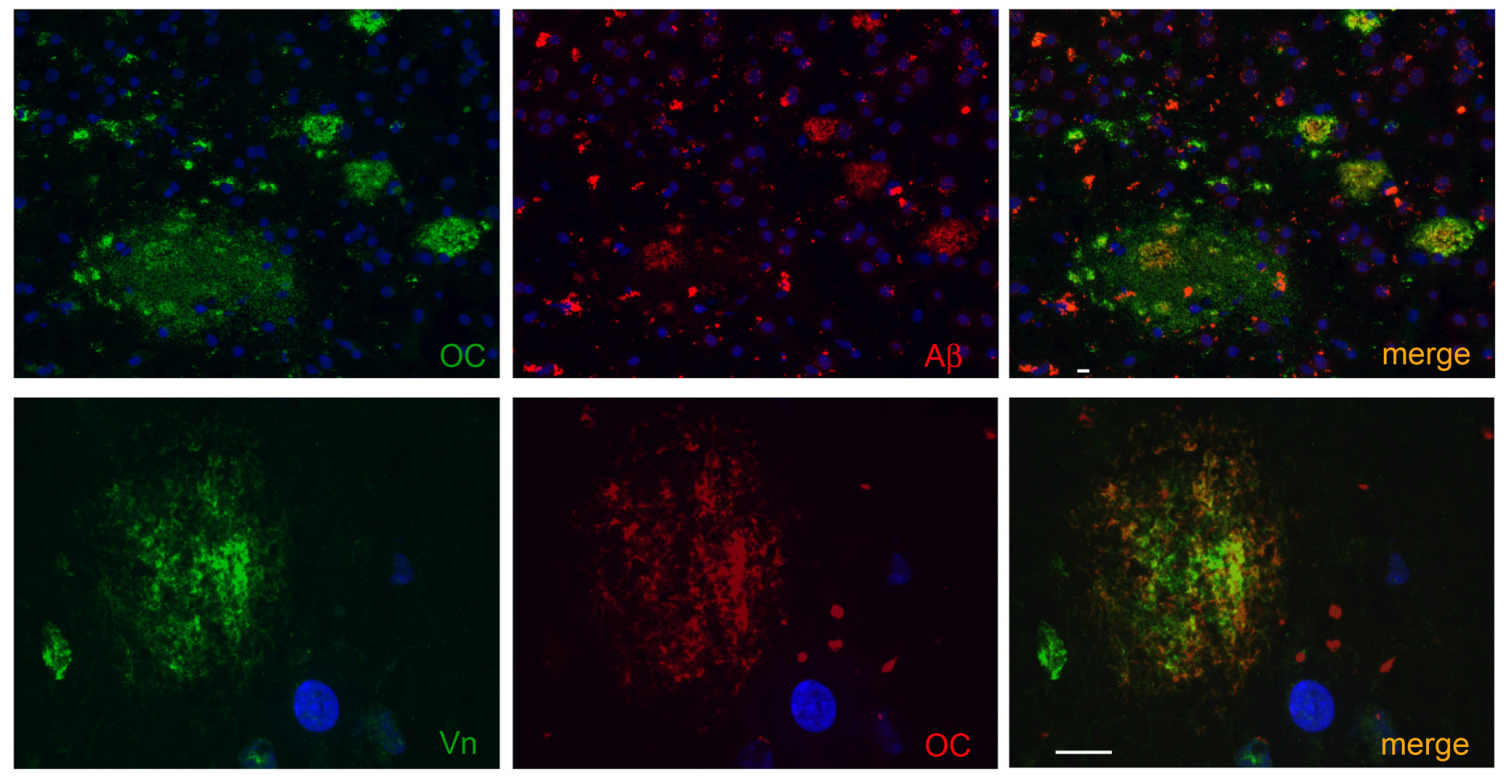
**Vitronectin deposits in senile plaques of Alzheimer disease brain**. *Upper panel*: senile plaques of AD brain visualized with the OC antibody (green) and the 6E10 antibody against Aβ (red). *Lower panel*: vitronectin (green) reactivity shows extensive overlap with OC reactivity (red), suggesting that vitronectin is deposited at sites of amyloid formation. Co-localization appears as yellow-orange color, while lipofuscin autofluorescence is seen predominantly in the red channel. Nuclei were visualized with DAPI (blue). Scale bar = 10 μm.

In order to assess the presence of vitronectin in extracellular deposits, we examined Alzheimer disease brain. In the senile plaques of AD brain, Aβ reactivity (Figure 1, upper panel, red) showed extensive overlap with the OC antibody (Figure 1 upper panel, green) which specifically recognizes amyloid fibrils and fibrillar oligomers. Lipofuscin autofluorescence is seen in the red channel together with Aβ but is not visible in the green channel. It can thus be distinguished by the red fluorescence. Similarly, robust vitronectin staining (Figure 1, lower panel, green) exhibited extensive overlap with OC reactivity in the plaques (Figure 1, lower panel, red). Overall, in the four cases of AD brain we examined there was a high correspondence of vitronectin reactivity in amyloid plaques, suggesting that vitronectin accumulates specifically in extracellular deposits that contain amyloid species.

### Vitronectin forms soluble nonfibrillar oligomers and amyloid fibrils

**Figure 2:**
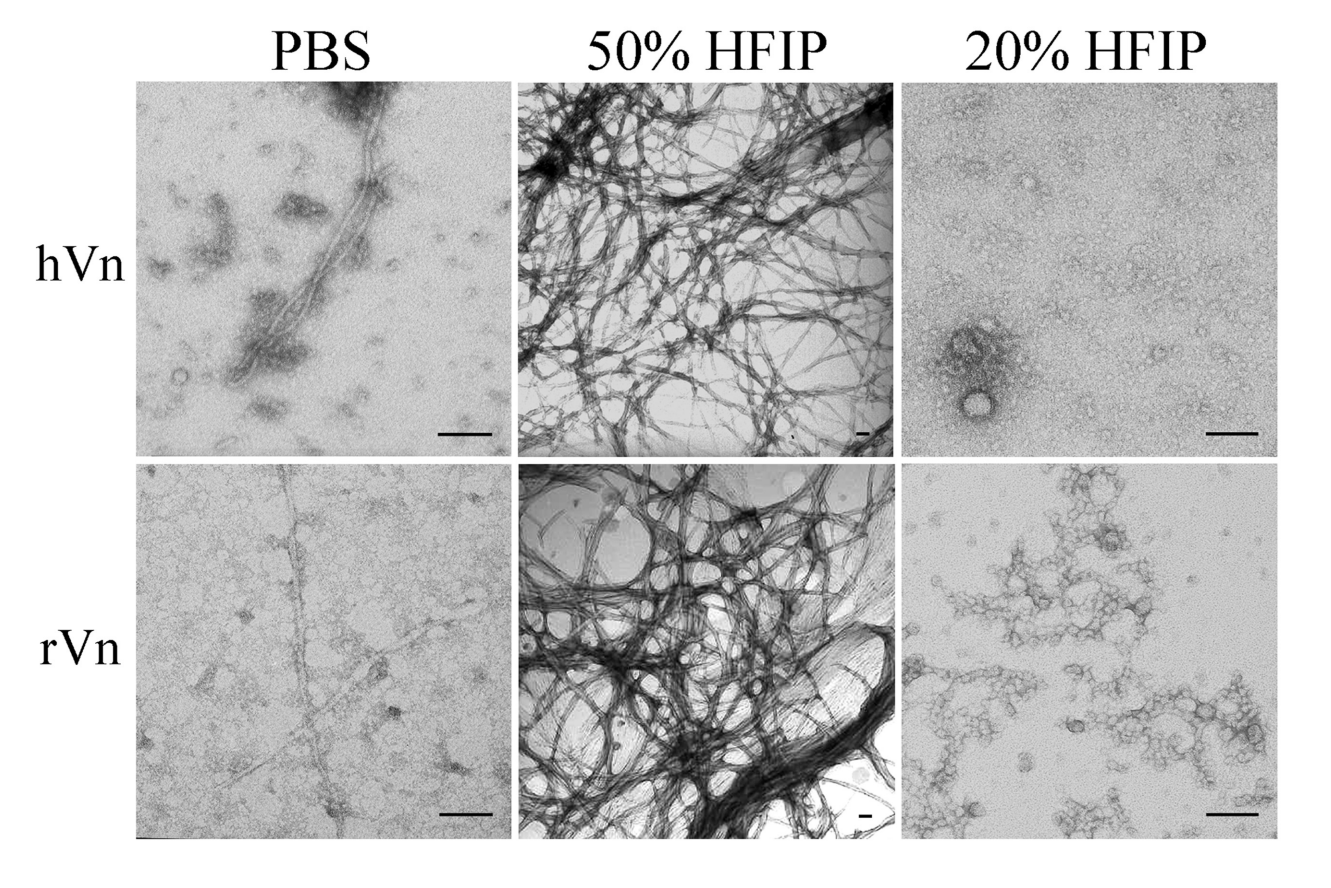
**Human vitronectin forms amyloid fibrils and oligomers**. *Left*, Plasma-purified (hVn) and recombinant human vitronectin (rVn) aged in phosphate-buffered saline form a heterogeneous mixture of spherical and fibrillar structures as seen under TEM. *Middle*, Vitronectin treated with 50% HFIP promotes the formation of typical amyloid fibrils that are approximately 3–8 nm in diameter. *Right*, Incubation in 20% HFIP and 1 mM HCl enriches the population of spherical and protofibrillar oligomers, which range from 6–35 nm in diameter. Scale bar = 100 nm.

To investigate whether vitronectin has an inherent propensity to behave as an amyloid protein, we aged plasma-purified and recombinant human vitronectin in phosphate-buffered saline (PBS). Plasma-purified and recombinant vitronectin in PBS form spherical and fibrillar structures by TEM (Figure 2, left). Because vitronectin in PBS produces a heterogeneous mixture of aggregates, we used 1,1,1,3,3,3-hexafluoro-2-propanol (HFIP), a commonly used solvent which enhances the rate of aggregation [52,53], to enrich the fibril and oligomer populations. Treatment with 50% HFIP promotes the formation of vitronectin fibrils, which resemble the morphology of typical amyloid fibrils (Figure 2, middle). The fibrils are approximately 3–8 nm in diameter, of varied length, and often twisted. Plasma-purified and recombinant vitronectin incubated in 20% HFIP and 1 mM HCl adopt spherical and protofibrillar structures, which range in size from 6–35 nm in diameter (Figure 2, right). Both vitronectin oligomers and fibrils enhance thioflavin T fluorescence (data not shown).

### A11-positive vitronectin oligomers are toxic to SH-SY5Y and RPE cells and induce membrane leakage

**Figure 3:**
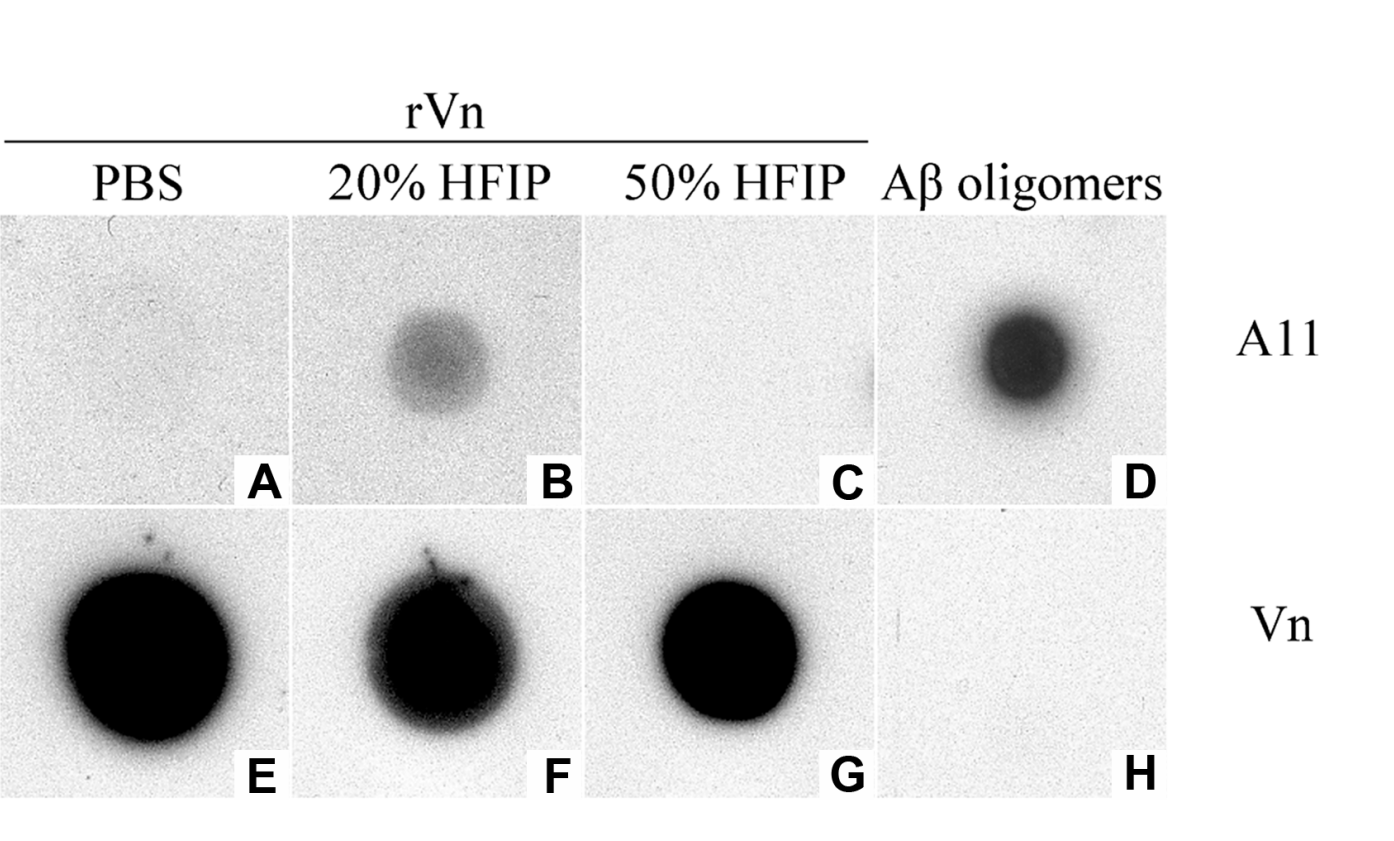
**Vitronectin forms soluble nonfibrillar amyloid oligomers**. Samples were spotted on a nitrocellulose membrane and probed with either A11 (A-D) or vitronectin (E-H) primary antibodies. Recombinant vitronectin (rVn) incubated in 20% HFIP and 1 mM HCl exhibits reactivity to the oligomer-specific A11 antibody (B), whereas vitronectin freshly dissolved in phosphate buffer (A) or treated with 50% HFIP (C) do not, suggesting that treatment with 20% HFIP enriches the population of soluble oligomers. Reactivity of samples with the vitronectin antibody confirms the presence of protein (E-G). Aβ oligomers served as a positive control for the A11 antibody (D), but are not detected by the vitronectin antibody (H).

To further characterize recombinant vitronectin oligomers, we performed a dot blot assay using the conformation-specific A11 antibody, which recognizes toxic oligomers formed by a variety of amyloidogenic proteins. Recombinant vitronectin freshly dissolved in phosphate buffer (Figure 3A) and vitronectin treated with 50% HFIP to induce fibrils (Figure 3C) are not recognized by the A11 antibody. However, recombinant vitronectin incubated in 20% HFIP and 1 mM HCl exhibits A11 immunoreactivity (Figure 3B), suggesting that the population of soluble nonfibrillar oligomers is enriched under these conditions. The quantity of vitronectin on the membrane was assessed using a primary anti-vitronectin antibody (Figure 3F–I). Aβ oligomers (Figure 3D) served as a positive control for the A11 antibody, but were not recognized by the vitronectin antibody, as expected (Figure 3H).

**Figure 4:**
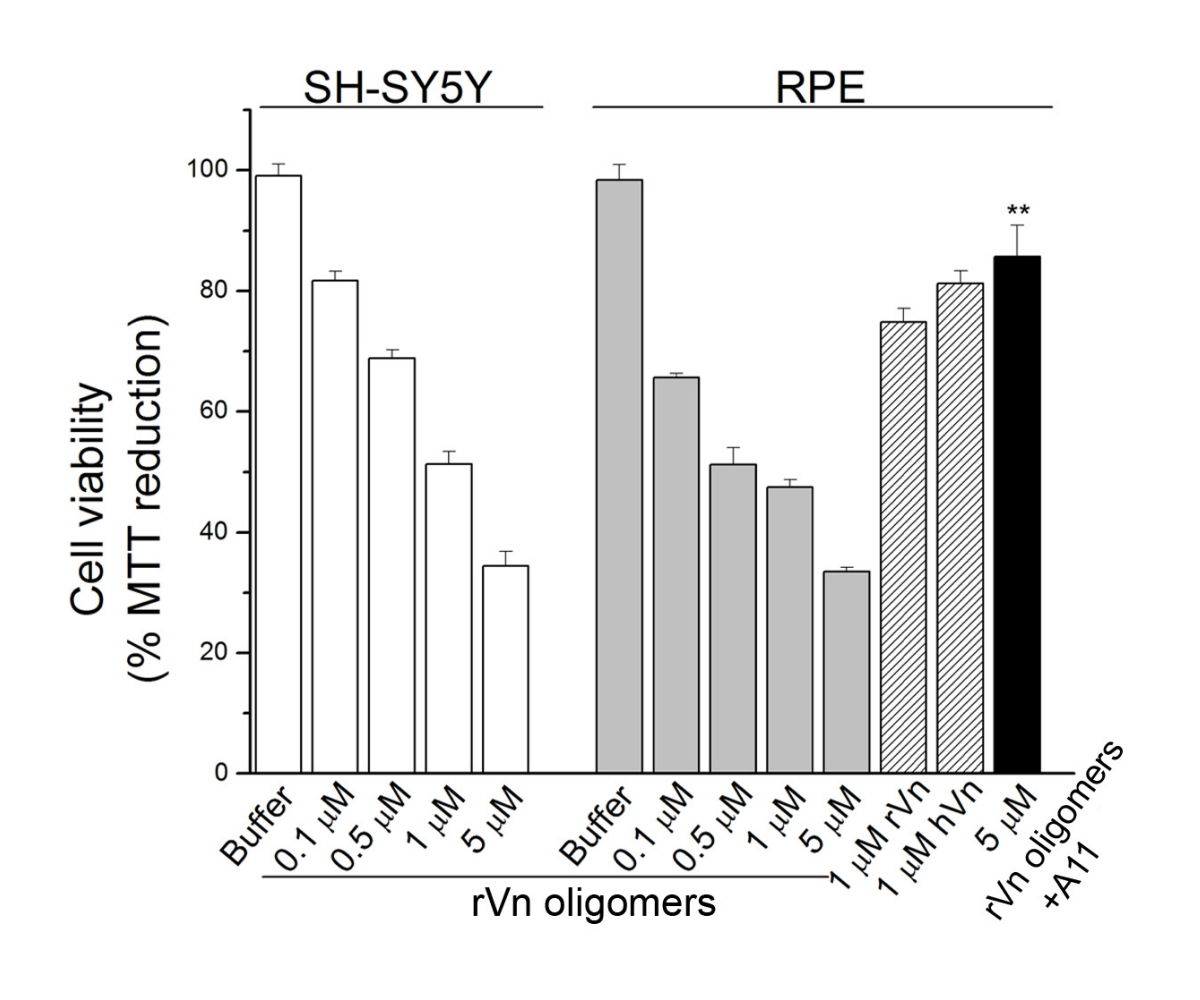
**Soluble nonfibrillar vitronectin oligomers compromise cell viability**. Recombinant vitronectin (rVn) oligomers are toxic to SH-SH5Y (white bars) and RPE cells (grey bars) in a dose-dependent manner, as evidenced by impaired MTT reduction. Plasma-purified (hVn) and recombinant vitronectin aged in PBS exhibit mild toxicity (hatched bars). RPE toxicity was significantly attenuated when 5 μM recombinant vitronectin oligomers were pre-incubated with an equimolar amount of A11 antibody prior to treatment (black bar), although complete rescue was not observed. Cell viability was assessed spectrophotometrically using an MTT-based assay. Error bars represent standard error of the mean. (n = 3; **p < 0.01).

Recent evidence suggests soluble nonfibrillar oligomers may comprise a toxic species. Because A11 immunoreactivity has been reported to correlate with cytotoxicity and recombinant vitronectin oligomers are A11-positive, we examined their effects on cultured SH-SY5Y and retinal pigment epithelium (RPE) cells. SH-SY5Y neuroblastoma cells and RPE cells were chosen due to the emerging role of soluble oligomers as pathogenic entities in neurodegenerative diseases such as Alzheimer disease and age-related macular degeneration. HFIP was removed by evaporation and indicated concentrations of recombinant vitronectin oligomers diluted in serum-free DMEM were added to cells for 4 hours at 37°C. Cell viability was assessed spectrophotometrically using an MTT-based assay. Vitronectin oligomers were toxic to both SH-SY5Y and RPE cells in a dose-dependent manner, as observed by impaired MTT reduction (Figure 4, white and grey bars). Plasma-purified and recombinant vitronectin aged in PBS exhibited mild RPE toxicity (Figure 4, hatched bars). Pre-incubation of 5 μM recombinant vitronectin oligomers with an equimolar amount of A11 antibody prior to treatment significantly attenuated the impairment in MTT reduction, indicating oligomer-specific toxicity (Figure 4, black bar) and demonstrating the ability of the A11 antibody to block the majority of toxic oligomeric species.

**Figure 5:**
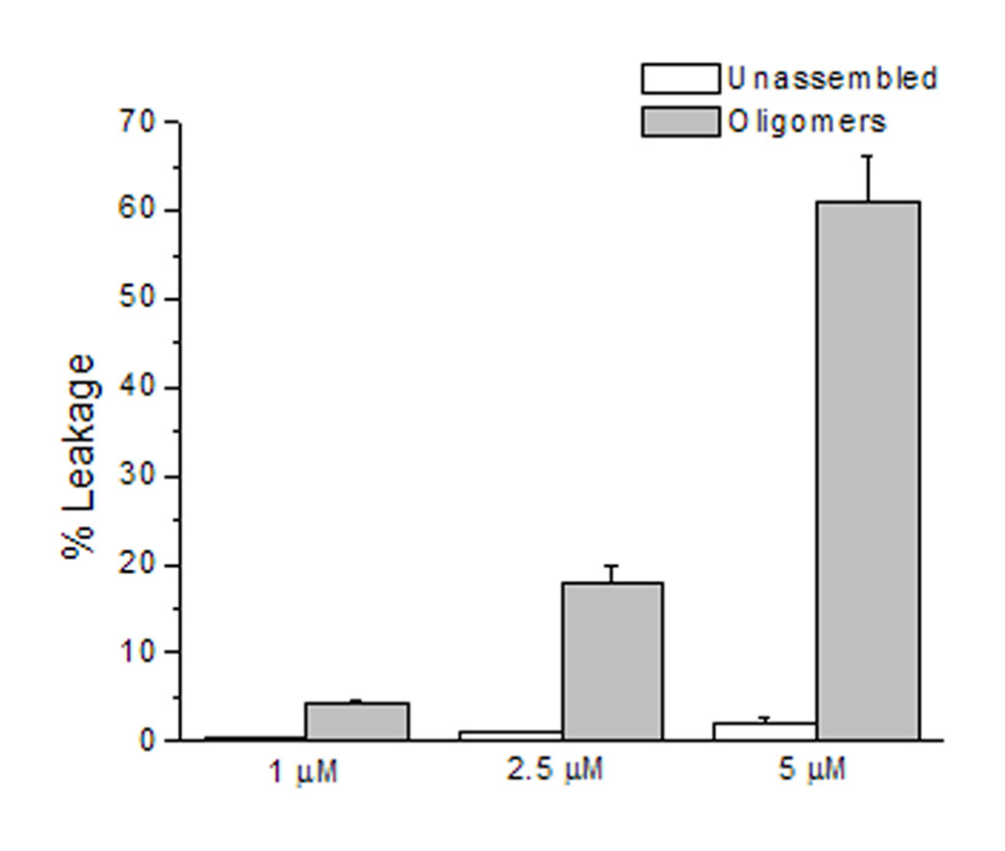
**Vitronectin oligomers induce membrane leakage**. Large unilamellar vesicles (100 nm in diameter) loaded with the fluorophore-quencher pair ANTS-DPX were incubated with indicated concentrations of protein. Recombinant vitronectin oligomers incubated with 500 μM LUVs resulted in appreciable leakage of vesicles in a dose-dependent fashion (grey bars) as compared to recombinant vitronectin freshly dissolved in phosphate buffer (white bars). Membrane leakage was assessed by monitoring ANTS fluorescence (λ_ex _= 353 nm, λ_em _= 520 nm). Intensities were normalized to the intrinsic fluorescence of the vesicles and percent leakage was estimated by dividing the sample and maximum fluorescence intensity values. Data shown are the combined average of three independent experiments. Error bars represent the standard error of the mean.

It has been suggested that the plasma membrane is a common target of toxic nonfibrillar oligomers. To investigate the hypothesis that vitronectin oligomers disrupt membrane integrity, we employed a fluorescence-based leakage assay using large unilamellar vesicles (LUVs) of 100 nm diameter loaded with the fluorophore-quencher pair ANTS-DPX. Indicated concentrations of freshly-dissolved recombinant vitronectin or recombinant vitronectin oligomers were diluted in Buffer 1 (see Materials and Methods) containing 500 μM lipids. ANTS fluorescence was monitored over time at 520 nm (λ_ex _= 353 nm) and percent leakage was estimated by dividing the sample and maximum fluorescence intensity values after correcting for the intrinsic fluorescence of the vesicles. Freshly-dissolved recombinant vitronectin had no appreciable effect on membrane permeability (Figure 5, white bars). Vitronectin oligomers induced dose-dependent leakage of ANTS from LUVs (Figure 5, grey bars), indicating that membrane disruption is caused by the oligomeric conformation.

### Trypsin digestion of vitronectin fibrils reveals a protease-resistant core

**Figure 6:**
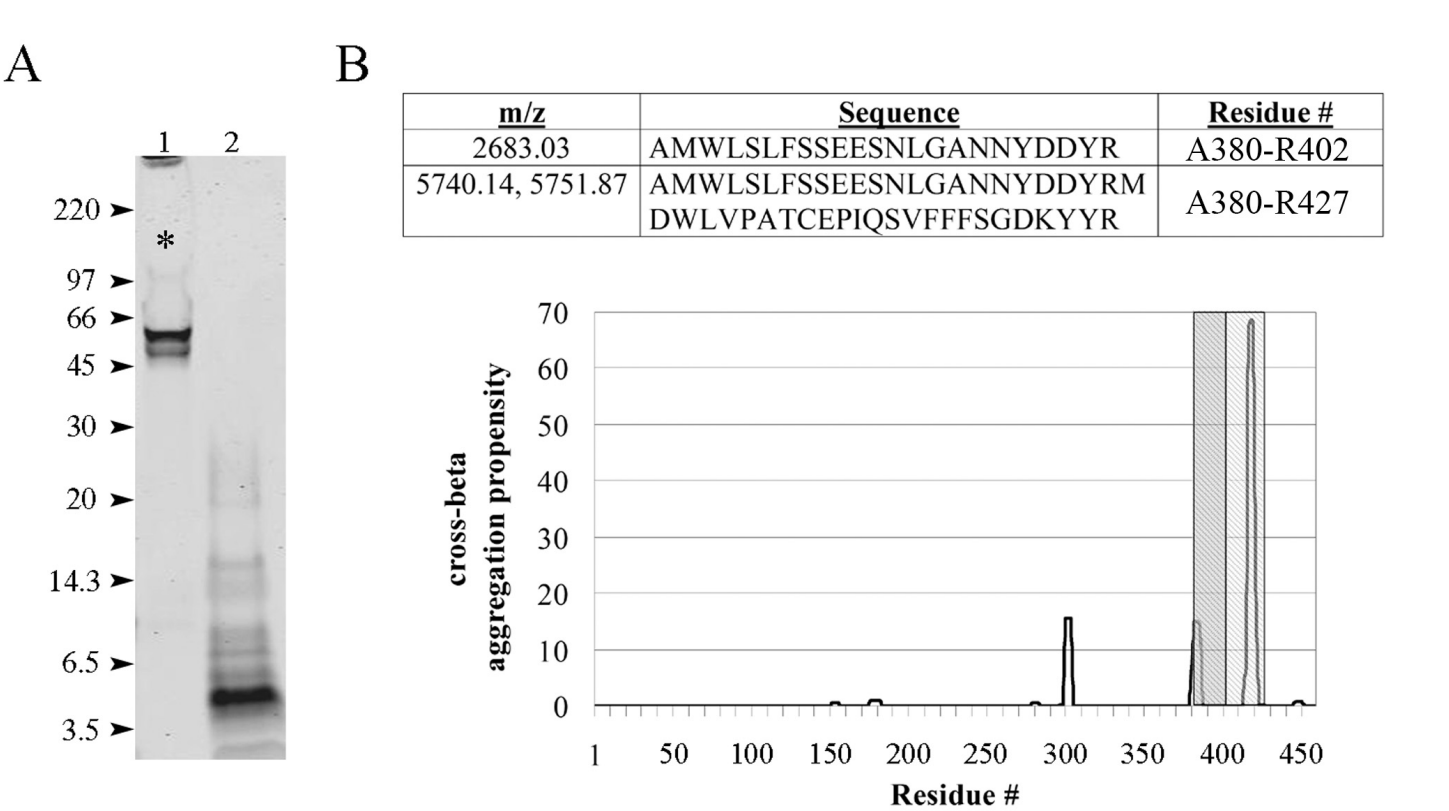
**Vitronectin fibrils contain a protease-resistant core**. A) In-solution trypsin digestion of vitronectin fibrils reveals a protease-resistant band of ~5 kDa (lane 2). Partial sequencing by N-terminal Edman degradation identified the peptide AMWLSLFSSEESNLGANNYDD, which corresponds to residues 380–400. These amino acids reside in the C-terminus of the protein and likely comprise a portion the amyloidogenic core. This result was confirmed by immunoblotting with a C-terminal-specific antibody (data not shown). Arrowheads indicate molecular weight (kDa). Lane 1 contains undigested vitronectin fibrils, much of which is unresolved (asterisk). B) *Upper panel*: Several peptides were identified by MALDI-TOF analysis of the protease-resistant band. The mass/charge (m/z) values, amino acid sequence and residue numbers are displayed in the table. Each m/z does not correspond to any other vitronectin peptides and oxidation had to be invoked for peptide A380-R427. *Lower panel*: Primary sequence analysis of vitronectin using the TANGO algorithm [68] reveals several C-terminal stretches prone to cross-beta aggregation, two of which correlate well with the peptides A380-R402 (gray) and A380-R427 (hatched) identified by mass spectrometry. Y-axis denotes relative cross-beta aggregation propensity.

To facilitate the isolation and identification of the amyloidogenic core of vitronectin fibrils, we employed protease digestion followed by peptide sequencing, immunoblotting, and mass spectrometry analysis. Undigested vitronectin fibrils are shown in Figure 6A (lane 1). It is evident that a large amount of aggregated protein was not resolved by SDS-PAGE and was retained in the wells or did not pick up the Coomassie stain (Figure 6A, asterisk). Trypsin digestion of vitronectin fibrils produced a major protease-resistant fragment of ~5 kDa (Figure 6A, lane 2). This band was not present when soluble recombinant vitronectin was digested (data not shown). Partial protein sequencing of the first twenty-one amino acids by N-terminal Edman degradation identified the peptide AMWLSLFSSEESNLGANNYDD in the excised band, which comprises C-terminal residues 380–400. The origin of the peptide was confirmed by western blot with a C-terminal-specific vitronectin antibody (data not shown). MALDI-TOF mass spectrometry analysis of the excised fragment identified several vitronectin peptides (Figure 6B) and suggests that residues 380–427 may be important for amyloid formation. The peptides correlate well with C-terminal stretches of increased cross-beta aggregation propensity as predicted by the TANGO algorithm (Figure 6B).

## Discussion

In this study, we demonstrate that in the AD brain, vitronectin and the fibril-specific OC antibody showed extensive overlap within senile plaques, suggesting that vitronectin may participate in amyloid formation at those sites. Importantly, we provide evidence that vitronectin is capable of amyloid formation. Consistent with previous reports [37-39], we observed that vitronectin readily forms aggregates, even in physiologic buffer. In this study we demonstrate that such aggregates display amyloid structures. These results extend previous *in vitro *studies of vitronectin aggregation and provide a possible structural basis for such oligomerization. HFIP treatment, although not required, enriched the individual populations of soluble, spherical oligomers and amyloid fibrils. Our results establish the amyloidogenicity of vitronectin and are in agreement with previously published reports regarding the toxicity of soluble oligomers [54]. The finding that HFIP treatment enhances oligomer and fibril formation supports the hypothesis that partial unfolding or denaturation of the native protein state promotes misfolding and subsequent formation of amyloid structures [55]. Vitronectin is known to accumulate in extracellular deposits in the eye and brain that are associated with macular degeneration and Alzheimer disease, respectively [4,21,24,27,33]. Interestingly, vitronectin oligomers recognized by the conformation-specific A11 antibody are cytotoxic to cultured RPE and SH-SY5Y cells in a dose-dependent manner. Soluble oligomers formed *in vitro *from a variety of proteins appear morphologically similar and exhibit toxicity to cultured cells [48,54,56-63], suggesting a common pathogenic mechanism. Pre-incubation of vitronectin oligomers with the A11 antibody prior to treatment significantly rescued RPE cell viability, demonstrating that the majority of toxic nonfibrillar oligomers are neutralized by the A11 antibody. Vitronectin oligomers permeabilize synthetic vesicles in a cell-free assay, which is consistent with studies showing that soluble amyloid oligomers disrupt membranes [52,62,64-67].

Vitronectin fibrils resemble the morphology of typical amyloid fibrils and contain a protease-resistant domain which likely contains the core residues sufficient for amyloid formation. The trypsin-resistant band was seen by SDS-PAGE and Coomassie stain when vitronectin fibrils were digested, but not when soluble recombinant vitronectin was digested. Sequencing and immunoblotting of the fragment revealed a C-terminal epitope and mass spectrometry analysis identified several peptides within residues 380–427, indicating that this region may be important for amyloid formation. Interestingly, the identified fragments overlap with two C-terminal regions that have increased cross-beta aggregation propensity, as calculated by the TANGO algorithm [68]. This region has been predicted to have a beta-propeller like domain in solution [69]. The combined data from mass spectrometry and TANGO analysis suggest the existence of an ordered amyloid core which limits protease accessibility to the stretch of amino acids between the two predicted aggregation-prone regions. The putative amyloid core resides within a naturally-occurring 10 kDa fragment (residues 380–459). Primary sequence analysis of the 10 kDa vitronectin fragment reveals a stretch of highly hydrophobic amino acids, which is consistent with the hypothesis that hydrophobicity is a key factor in aggregation propensity [55,68,70-72]. Further studies are warranted to precisely delineate the boundaries of the vitronectin amyloidogenic core and to characterize the *in vitro *and *in vivo *significance of the 10 kDa vitronectin fragment.

Vitronectin exists in two distinct conformations *in vivo*. The majority of vitronectin in plasma and serum circulates as a non-heparin-binding monomer. Approximately 2–8% of vitronectin in the blood is in the alternate heparin-binding, partially unfolded conformation which can self-associate [73]. This multimeric form is thought to be the predominant conformation of vitronectin in the extracellular matrix [74]. Chaotropic denaturation, which was used in the purification of recombinant vitronectin, exposes the heparin-binding site [37]. Since vitronectin readily aggregates in its heparin-binding state, this specific conformation may aid in amyloid formation, a hypothesis further supported by the observation that hydrophobic interactions appear to drive functional oligomerization [75]. Interestingly, the protease-resistant region is located adjacent to the heparin-binding domain.

## Conclusion

Although a relatively small percentage of vitronectin is in an alternate conformation, conditions of high local concentration via increased synthesis or recruitment, which may occur at extravascular sites or in the setting of chronic inflammation, may promote vitronectin misfolding and amyloid formation. Our results put forth the possibility that vitronectin misfolding and amyloid formation may contribute to age-related diseases such as atherosclerosis, AMD, and AD.

## Methods

### Antibodies and reagents

Plasma-purified vitronectin was purchased from Biosource (Camarillo, CA). The 6E10 antibody against beta-amyloid (Aβ) 1–16 was purchased from Covance Research Products, Inc. (Dedham, MA). The anti-oligomer A11 antibody and OC antisera were generated as previously described [48,76]. Production of a polyclonal anti-vitronectin antibody raised against full-length recombinant vitronectin was performed by Biomer Technology (Hayward, CA). The IgG fraction was enriched from rabbit serum using Affi-Gel Protein A support (Bio-Rad; Hercules, CA). Secondary antibodies were purchased from Vector Laboratories (Burlingame, CA). All other reagents were purchased from Sigma-Aldrich (St. Louis, MO), unless otherwise specified.

### Immunocytochemistry

Frozen human cortex was obtained from the USC Alzheimer Disease Research Center. Intact human eyes were obtained from the Oregon Lions Sight and Hearing Foundation. Tissue was prepared as described previously [35]. Briefly, sections were blocked overnight in phosphate-buffered saline containing 2% BSA and 2% goat serum, followed by incubation with either the anti-vitronectin antibody or OC antisera. Sections were washed and incubated with either FITC- or Texas Red-conjugated goat anti-rabbit antibody. DAPI was used to visualize nuclei. Images were acquired on an UltraVIEW VoX spinning disk confocal microscope (PerkinElmer, Waltham, Massachusetts).

### Cloning, expression, and purification of full-length human vitronectin

A plasmid containing human vitronectin cDNA was purchased from American Tissue Culture Company (ATCC; Rockville, MD) and a 1,385 bp fragment of mature, full-length human vitronectin cDNA containing engineered N-terminal NcoI and C-terminal HindIII restriction sites was synthesized by polymerase chain reaction. The fragment was digested with NcoI and HindIII restriction enzymes (New England Biolabs; Ipswich, MA) and subcloned into a pSE420 vector with complementary sticky ends. Correct orientation of the insert was verified by DNA sequencing and *Escherichia coli *DH5α cells were transformed with the 4.27 kb construct. Cells were grown with shaking at 37°C to an optical density of 0.6–0.8 and protein expression was induced by the addition of 1 mM isopropyl-1-thio-β-D-galactopyranoside (IPTG) for 4 hours at 37°C. Cells were harvested by centrifugation at 5,000 × g for 15 minutes and the pellet was stored at -80°C. Vitronectin protein was purified using a previously described protocol [77] with modifications. The pellet was resuspended in lysis buffer [5 mM EDTA, 10 mM dithiothreitol (DTT), 1 mg/mL lysozyme, protease inhibitors, all in 10 mM sodium phosphate buffer, pH 7.4], incubated on ice for 1 hour, then nutated at 4°C for 10 minutes with the addition of 24 U/ml DNase I, 60 mM MgCl_2_, and 1% Triton X-100. The suspension was sonicated 3 × 1 minute on ice, with one minute between sonications, and centrifuged at 20,000 × *g *for 30 minutes. The pellet was resuspended in 2 M urea buffer (2 M urea, 5 mM EDTA, 10 mM DTT, protease inhibitors, all in 10 mM sodium phosphate buffer, pH 7.4), sonicated on ice, centrifuged, and supernatant decanted. The resulting pellet of inclusion bodies was solubilized in cold 8 M urea buffer (8 M urea, 5 mM EDTA, 10 mM DTT, protease inhibitors, all in 10 mM sodium phosphate buffer, pH 7.4), sonicated on ice, and centrifuged. The supernatant was applied to a HiTrap heparin affinity column (Amersham Biosciences; Piscataway, NJ) equilibrated with 8 M urea buffer and proteins were eluted using a linear NaCl gradient. Vitronectin-containing fractions were identified by SDS-PAGE, pooled, and dialyzed against 1% acetic acid, 10 mM phosphate buffer (pH 7.4), or PBS. Protein purity was >95% by Coomassie Blue staining. Protein concentration was calculated from absorbance at 280 nm (ε_M _= 82,330 M^-1 ^cm^-1^).

### Fibril and oligomer preparation

Lyophilized vitronectin protein in a siliconized microcentrifuge tube was dissolved in cold 1,1,1,3,3,3-hexafluoro-2-propanol (HFIP) for 10 minutes at room temperature. For fibrils, this solution was diluted to 50% HFIP in water and stirred at room temperature for 7–14 days at a protein concentration of 10–25 μM. Fibril formation was monitored by transmission electron microscopy (TEM). To prepare soluble oligomers, vitronectin in HFIP was diluted to 20% HFIP and 1 mM HCl and stirred at room temperature with a vented lid for 3–7 days. The final protein concentration was approximately 20 μM after the gradual evaporation of HFIP. Oligomer formation was monitored by TEM and by dot blot using the anti-oligomer antibody. Alternatively, lyophilized vitronectin was resuspended in PBS and aged for 2–4 weeks at room temperature with or without stirring to form oligomers and fibrils without HFIP.

### Electron microscopy

HFIP was evaporated under a gentle stream of nitrogen. Ten microliters of sample was applied to a 200-mesh formvar-coated nickel grid (Electron Microscopy Sciences; Hatfield, PA) for 5 minutes, stained in 3% uranyl acetate for 5 minutes, rinsed, and air-dried. The grids were examined using a Jeol JEM1200EX microscope at 80 kV.

### Dot blot

Two microliters of each sample were spotted onto nitrocellulose membrane and allowed to air-dry. Tris-buffered saline (20 mM Tris, 0.8% NaCl, pH 7.4) containing 0.001% Tween-20 (TBST) was used for washing and dilution. The membrane was blocked for 1 hour with 10% nonfat dried milk in TBST, washed 3 × 10 minutes, incubated for one hour in primary anti-oligomer antibody (1:5,000 in 3% BSA/TBST), washed 3 × 10 minutes, incubated for 30 minutes in HRP-conjugated secondary anti-rabbit antibody (1:10,000 in 3% BSA/TBST), and washed 3 × 10 minutes. The membrane was developed using enhanced chemiluminescence reagents (Amersham Biosciences; Piscataway, NJ) and exposed to Hyperfilm (Amersham Biosciences; Piscataway, NJ). The same procedure was performed for dot blot with anti-vitronectin antibody (1:10,000; Biomer Technology, Hayward, CA) using 0.1% Tween-20 in TBST. A control membrane with primary antibody omitted was simultaneously processed.

### Cell culture

SH-SY5Y cells were obtained from ATCC (Rockville, MD). Retinal pigment epithelium (RPE) cells were isolated from human fetal eyes obtained from Advanced Bioscience Resources, Inc. (Alameda, CA) as described previously [78]. Cells were maintained in complete medium [Dulbecco's modified Eagle's medium (DMEM, VWR; West Chester, PA) supplemented with 2 mM L-glutamine, 100 U/ml penicillin, 100 μg/ml streptomycin, and 10% fetal bovine serum (Invitrogen; Carlsbad, CA)] at 37°C in a humidified incubator. Fourth-passage cells were seeded in a 96-well plate at 2 × 10^4 ^cells per well and grown in complete medium for 3–4 days to approximately 90% confluence. Cells were maintained in serum-free medium for one day prior to the experiment. On the day of the assay, media was removed, replaced with the indicated samples diluted in DMEM, and incubated at 37°C for 4 hours. MTT (3-[4, 5-dimethylthiazol-2-yl]-2, 5-diphenyl tetrazolium bromide) dissolved in DMEM was added and cells were placed at 37°C for an additional 4 hours. Tetrazolium crystals were dissolved by the addition of MTT solubilization solution (10% Triton X-100, 0.1 N HCl in anhydrous isopropanol), and absorbance was measured at 570 nm. Experiments were carried out in triplicates.

### Membrane leakage

Large unilamellar vesicles (LUVs,100 nm diameter) containing 90% phosphatidylcholine and 10% phosphatidylserine (Avanti Polar Lipids; Alabaster, AL) were extruded in the presence of the fluorophore-quencher pair 8-aminonaphthalene-1,3,6-trisulfonic acid (ANTS) and p-xylene-bis-pyridinium bromide (DPX). To assess membrane leakage, a 250 μl solution containing 500 μM LUVs and the sample of interest in Buffer 1 (10 mM HEPES, 50 mM KCl, 1 mM EDTA, 3 mM sodium azide) was placed in a 2 mm path-length quartz cuvette. ANTS fluorescence was monitored as a function of time at 520 nm with excitation at 353 nm using a Jasco FP-6500 spectrofluorimeter. Maximum fluorescence intensity was determined by adding 5 μl of 10% Triton X-100. Intensities were normalized to the intrinsic fluorescence of the vesicles and percent leakage was estimated by dividing the sample and maximum fluorescence intensity values. The data represent the results of three independent experiments.

### Protease digestion

Fibrils were collected by centrifugation and the pellets were digested in a solution containing 1:25 trypsin (protease-to-protein mass ratio) and 10 mM DTT, all in 50 mM ammonium bicarbonate, pH 8, at 37°C. Overnight digests were heat inactivated at 60°C, analyzed by SDS-PAGE, and visualized with Coomassie Blue staining. The major protease-resistant fragment was either transferred onto a PVDF membrane and submitted to the USC/Norris Microchemical Core Facility for peptide sequencing by N-terminal Edman degradation, or excised and passively eluted in 50 mM Tris, 50 mM NaCl (pH 8) and submitted to the USC Proteomics Core Facility for matrix-assisted laser desorption/ionization time-of-flight (MALDI-TOF) mass spectrometry analysis.

## Competing interests

CG and RK are paid consultants for Kinexis, Inc.

## Authors' contributions

TMS carried out the molecular cloning, protein purification, preparation of fibrils and oligomers, dot blot, cell culture and toxicity assays, protease digestion, mass spectrometry, data analysis, and drafted the manuscript. JMI participated in the electron microscopy, mass spectrometry, immunocytochemistry on human AD tissue, and data analysis. CH performed the immunocytochemistry on human AD tissue. RK and CGG developed and characterized the OC and A11 antibodies. RL and JC participated in concept, design, data analysis and manuscript preparation. All authors read and approved the final manuscript.
